# Variant Histone H2A.Z Is Globally Localized to the Promoters of Inactive Yeast Genes and Regulates Nucleosome Positioning

**DOI:** 10.1371/journal.pbio.0030384

**Published:** 2005-11-01

**Authors:** Benoît Guillemette, Alain R Bataille, Nicolas Gévry, Maryse Adam, Mathieu Blanchette, François Robert, Luc Gaudreau

**Affiliations:** **1** Centre de Recherche sur les Mécanismes du Fonctionnement Cellulaire, Département de Biologie, Faculté des Sciences, Université de Sherbrooke, Sherbrooke, Québec, Canada,; **2** Laboratoire de Chromatine et Expression du Génome, Institut de Recherches Cliniques de Montréal, Montréal, Québec, Canada,; **3** McGill Center for Bioinformatics, Lyman Duff Medical Building, Montréal, Québec, Canada; Fred Hutchinson Cancer Research CenterUnited States of America

## Abstract

H2A.Z is an evolutionary conserved histone variant involved in transcriptional regulation, antisilencing, silencing, and genome stability. The mechanism(s) by which H2A.Z regulates these various biological functions remains poorly defined, in part due to the lack of knowledge regarding its physical location along chromosomes and the bearing it has in regulating chromatin structure. Here we mapped H2A.Z across the yeast genome at an approximately 300-bp resolution, using chromatin immunoprecipitation combined with tiling microarrays. We have identified 4,862 small regions—typically one or two nucleosomes wide—decorated with H2A.Z. Those “Z loci” are predominantly found within specific nucleosomes in the promoter of inactive genes all across the genome. Furthermore, we have shown that H2A.Z can regulate nucleosome positioning at the *GAL1* promoter. Within HZAD domains, the regions where H2A.Z shows an antisilencing function, H2A.Z is localized in a wider pattern, suggesting that the variant histone regulates a silencing and transcriptional activation via different mechanisms. Our data suggest that the incorporation of H2A.Z into specific promoter-bound nucleosomes configures chromatin structure to poise genes for transcriptional activation. The relevance of these findings to higher eukaryotes is discussed.

## Abstract 2

A high-resolution ChIP-on-chip mapping of H2A.Z across the yeast genome suggests a relationship to transcriptional activity of the genes with which it is associated.

## Introduction

Eukaryotic chromosomes are packaged into a complex nucleoprotein structure termed chromatin, from which the fundamental unit is the nucleosome. Nucleosomes are composed of octamers of histone proteins designated as H2A, H2B, H3, and H4 that wrap approximately 146 bp of DNA [1,2]. Chromatin is generally highly repressive to cellular processes that involve DNA transactions such as gene transcription [3]. Cells have thus devised at least three schemes to overcome the nucleosomal barrier: the first involves adenosine triphosphate hydrolysis to mechanically displace nucleosomes [4,5], and the second consists of chemically modifying the tails of histones, such as by acetylation, phosphorylation, methylation, and ubiquitination [2]. This last scheme can take the form of a “code” where modifications act as signals for subsequent chromatin modifications [6,7]. A third scheme that has been devised by cells to overcome the nucleosomal barrier is to alter the composition of nucleosomes through the incorporation of histone variants that can—directly or indirectly—alter the permissiveness of chromatin to gene expression [8,9].

Nonallelic variants have been described for many classes of histones, and one of the best-studied examples is the Z variant of H2A. H2A.Z, formerly called H2A.F/Z, is evolutionarily conserved from yeast to mammals [10,11]. H2A.Z is essential for the viability of *Tetrahymena, Drosophila,* and mice [12–14] and constitutes approximately 10% of all cellular H2A molecules in S-phase [15,16]. Experiments carried out in many organisms have shown that H2A.Z has a function distinct from the canonical H2A [13,17,18]. H2A variant histones differ mainly in their N- and C-terminal portions as compared to S-phase H2A, whereas the core region is highly conserved [16]. In fact, experiments carried out in *Drosophila* and in yeast have revealed that the unique feature of H2A.Z important for its specific function resides in the carboxyl-terminal region of the variant histone and not in its histone fold [18,19].

In *Tetrahymena thermophila,* H2A.Z has been found to be associated with the transcriptionally active macronucleus, thus implying a positive role for H2A.Z in gene transcription [20]. Subsequently, experiments carried out in yeast have shown that H2A.Z could regulate transcription [19,21] and that its function is partially redundant with nucleosome remodeling complexes such as SAGA and Swi/Snf [21]. H2A.Z has been shown to be quickly remodeled soon after gene induction [19,21–23], a result that implies that the role of H2A.Z in positive gene transcription occurs at an early step. In line with this idea, we have shown that the transcriptional machinery is not efficiently recruited to the *GAL1* promoter in *htz1*Δ cells [19]. Another mechanism by which H2A.Z could contribute to gene regulation has been suggested by Farris et al. [23], where they have shown at the *c-myc* gene that transcription-induced chromatin transactions involve the exchange of H2A.Z for canonical H2A.

In addition to its well-established role in transcriptional activation, a recent important piece of work by the Madhani group showed that H2A.Z positively regulates clusters of genes that are proximal to telomeres [24]. The gene expression defect observed at those gene clusters (called HZAD domains) in H2A.Z mutants can be suppressed by the deletion of silencing proteins such as Sir2. It has therefore been suggested that H2A.Z functions as an antisilencing factor by antagonizing the spread of heterochromatin to euchromatic regions [24]. The expression of other H2A.Z-affected genes, however, is not sensitive to Sir proteins suggesting H2A.Z positively regulates gene expression via both antisilencing and other mechanisms.

Evidence also suggests that H2A.Z can negatively regulate gene expression, notably through gene silencing. In *Drosophila* and in yeast, H2A.Z was reported to be involved in heterochromatic silencing [25,26]. In mammalian cells, H2A.Z colocalizes with, and directly interacts with, heterochromatin protein 1 or HP1-α (LocusLink:23468) and with INCENP (LocusLink:3619)—a protein involved in chromosome segregation—in pericentric heterochromatin [27,28]. In addition, HP1-α and H2A.Z may function together to establish a specialized conformation at constitutive heterochromatin [27]. Thus, from these findings it is clear that H2A.Z is important both for the positive and negative regulation of gene expression.

To add to all of these recent findings, there has been the codiscovery of an adenosine triphosphate-dependent chromatin remodeling complex that specifically loads H2A.Z into chromatin and exchanges it with H2A [29–31]. This complex, from which the catalytic subunit is Swr1, also shares essential subunits with the NuA4 histone acetyltransferase complex [29]. In addition to their importance in gene regulation, the Swr1 complex, H2A.Z, and NuA4 are all involved in the regulation of chromosome stability in yeast [32]. This is interesting and important since it was also demonstrated that in mammalian cells, knockdown of H2A.Z causes the genome to become unstable, a process that is attributed to defects in chromosome segregation [33]. Of equal importance is the fact that H2A.Z is required for early mouse and *Xenopus* development [13,14].

Despite all the work reviewed above, the mechanisms by which H2A.Z regulates transcription, silencing, genome stability, and developement remains largely unknown. A key piece of data that is missing is a global view of the distribution of H2A.Z across the genome. Polytene chromosome stainings have shown that *Drosophila* H2A.Z is widely distributed in the genome, but with nonuniform distribution that is characterized by a complex banded pattern [34]. From these data, it is thus conceivable that certain regions of the genome are preferentially occupied by variant histones and interspersed with regular histones. The resolution provided by this assay, however, is not high enough to allow one to correlate H2A.Z localization with its effect on gene expression. Experiments aimed at localizing proteins on defined chromatin segments in vivo—chromatin immunoprecipitation (ChIP)—have been performed at specific loci, but these experiments do not allow determining whether H2A.Z assembles preferentially within large chromosomal domains, whether it is randomly spread out across the genome, or whether it is associated with specific loci along chromosomes. Answering this question is a crucial step toward understanding the various functions of this important protein.

Here we make use of a genome-wide location assay (ChIP–chip) to map H2A.Z across the yeast genome with a 300-bp resolution. Our data show that H2A.Z preferentially and specifically associates to small regions within the promoters of inactive yeast genes. We also show that replacement of H2A.Z for regular H2A within the *GAL1* promoter region perturbs nucleosome positioning, a situation that might account for the transcription defect at the latter gene in the absence of H2A.Z. Interestingly, the very strict promoter-specific association of H2A.Z that is otherwise observed elsewhere is not observed for genes contained within the HZAD domains. At those genes, the presence of H2A.Z can be detected within wider domains encompassing both the promoter region and part of the open reading frame (ORF).

## Results

### Genome-Wide Location Analysis of H2A.Z

In order to investigate the genomic distribution of H2A.Z in yeast, we performed genome-wide location experiments (also known as ChIP–chip) on whole-genome tiling microarrays. The assay consists of first performing ChIP assays to immunoprecipitate (IP) our target DNA molecules of interest. Thus, yeast cells are first cross-linked with formaldehyde prior to being lysed. Chromatin fragments (approximately 400 bp) are next obtained by sonication and specific antibodies are then used to IP our protein or proteins of interest along with their target DNA. Purified DNA is next amplified by ligation-mediated PCR (LM-PCR), labeled, and hybridizing to an array that contains a total of 44,290 60-mer probes (including controls) for an average of one probe every 266 bp (see Materials and Methods for more details). Thus, ChIPs were performed using yeast strains bearing Myc epitopes on H2A.Z and on the canonical H2A. In every case, a ChIP for histone H2B (performed using an HA-tagged H2B strain) was hybridized together with the H2A.Z (or H2A) ChIP to control for nucleosome density. In addition, other control experiments where two Myc-H2A.Z ChIPs are hybridized against each other were also performed. All experiments were performed in duplicate and combined using a weighted average method. A Gaussian filter was then applied to each dataset in order to create a smooth interpolation occupancy curve. The method allowed for the identification of 4,862 loci enriched in H2A.Z, referred to below as “Z loci.” The enrichment ratio for every probe on the microarray is available in Table S1, whereas the smoothed data can be found in Tables S2–S4. The coordinates of all Z loci are listed in Table S5. All datasets were deposited into ArrayExpress under accession number E-MEXP-396.

### H2A.Z Is Associated with Small Genomic Regions Present All across the Yeast Genome

**Figure 1:**
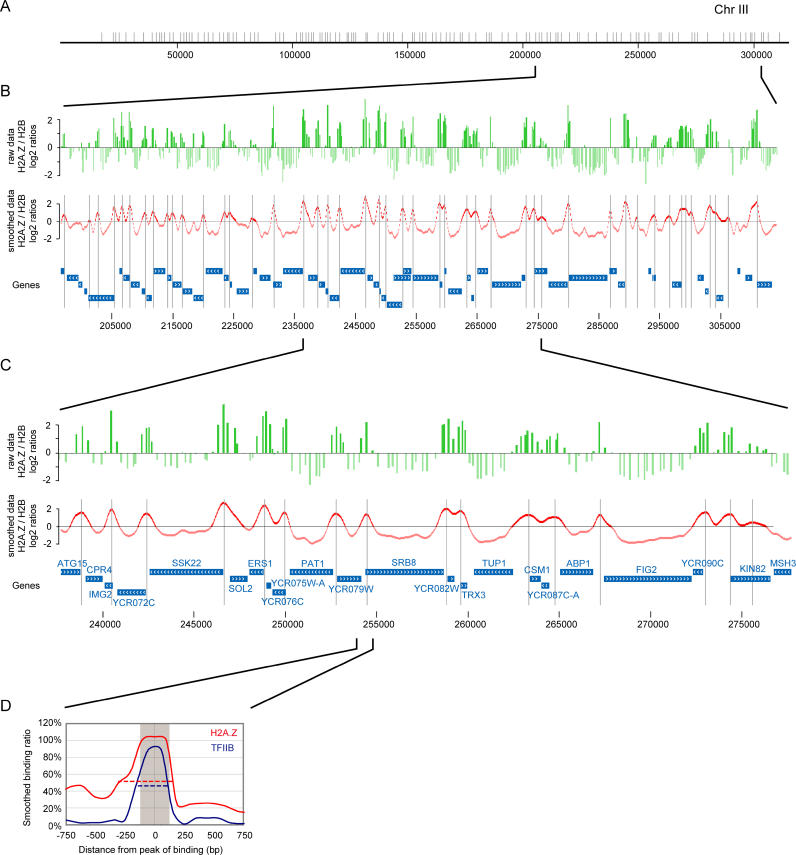
Genome-Wide Location Analysis of H2A.Z and H2A; a Zoom on Chromosome III (A) The location of the Z loci along Chromosome III is depicted by gray bars. (B) A zoom in a 120 KB region between position 196611–316611 shows the raw H2A.Z/H2B log_2_ ratios for each probe in that region (green bars) and the smoothed data resulted from the Gaussian plot analysis of the raw data for H2A.Z/H2B log_2_ ratios (red line). The position of the Z loci is shown by gray lines. The genes present in that regions are shown in blue. (C) Same as in (B) but for a zoom in region 237700–277700 (40 KB). (D) The size of the Z loci within the promoter of the *SRB8* gene as determined by Q-PCR analysis of ChIP experiments. The binding ratios for H2A.Z (red) and TFIIB (blue) are shown relative to the center of the probe that generates the maximum enrichment. The data were smoothed by a sliding median over three probes (see Figure S1). The size of the observed H2A.Z domain is about 250 bp larger than that of TFIIB. Since TFIIB covers about 10 bp of DNA, we can infer that H2A.Z covers about 260 bp of DNA at that locus (shaded area). More details can be found in Protocol S1 and Figure S1.

The 4,862 loci identified here are widely distributed across all chromosomes, and no regions show any bias (see Figure 1 for a map of Chromosome III and Table S6 to see the whole genome). Typical Z loci are less than 400 bp long, suggesting that the variant histone occupies regions that are generally no larger than two nucleosomes. This estimation is based on four observations. First, the probes enriched in the H2A.Z experiments were not depleted in the H2A experiments (data not shown). Because the average size of the DNA fragments used in our ChIP assays is 400 bp, this suggests that most 400-bp fragments within the genome that contains H2A.Z will also contain H2A. Since, in principle, a nucleosome cannot contain both H2A and H2A.Z simultaneously [35], the data suggest that H2A.Z occupies regions that are smaller than 400 bp. Second, the size of the Z loci as they appear from the microarray data (see Figure 1, for example) are consistent with H2A.Z occupying small regions—especially considering the fact that ChIP experiments necessarily overestimate the size of the region bound by proteins due to the size of the chromatin fragments generated. Third, high-resolution quantitative PCR (Q-PCR) analysis of ChIP experiments using small overlapping PCR probes allowed us to map the Z loci within the promoter of the *SRB8* genes to an approximately 260-bp region using TFIIB as a reference [36] (Figure 1D; see Protocol S1 and Figure S1 for more details). Last, alignment of our data with global nucleosome positioning information from Yuan et al. [37] revealed that H2A.Z maps to one or two nucleosomes in promoters (see below). Taken together, these analyses show that H2A.Z occupies very small regions scattered across the genome, rather than concentrating within large chromosomal domains.

### H2A.Z-Containing Nucleosomes Are Essentially Located within Promoter Regions

**Figure 2:**
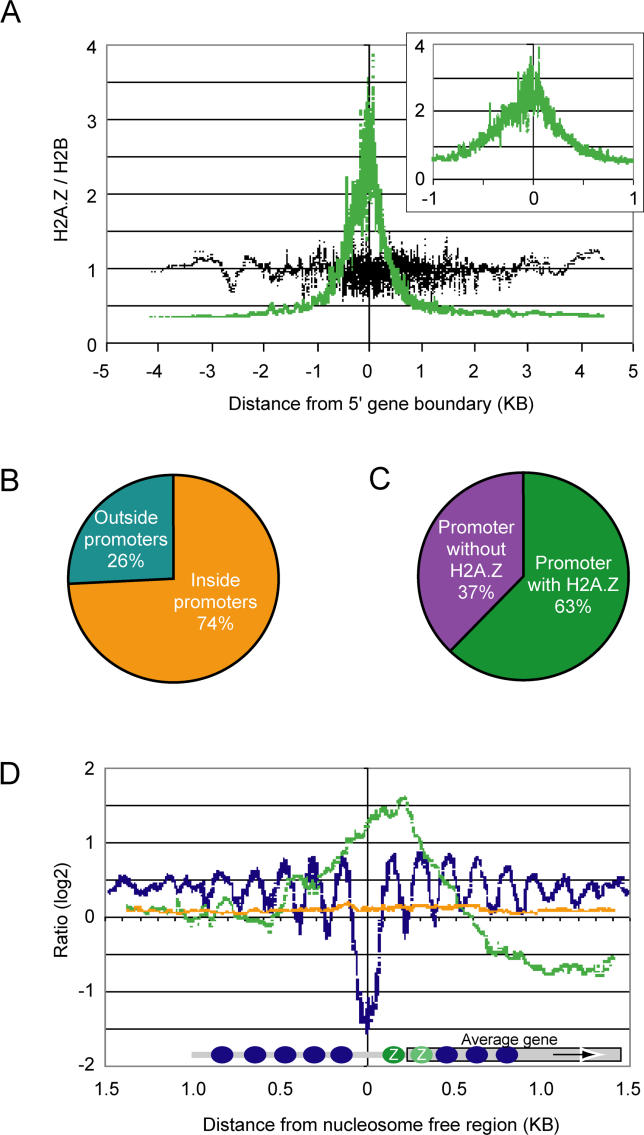
H2A.Z Is Predominantly Localized within Promoter Regions (A) A sliding median of the H2A.Z/H2B ratio for all probes on the microarray is plotted against the distance from the 5′ boundary of their closest transcribed element (including ORF, tRNA, transposon, etc.) (green). Randomized data (where the H2A.Z/H2B ratios were scrambled prior to calculating the sliding median) are plotted in black. (B) Most Z loci are found within promoters. The fraction of the 4,862 Z loci present within promoters (gold) and nonpromoter region (aqua) is shown. Promoter regions are defined as the −500/+100 interval relative to the 5′ boundary of transcribed elements as annotated in the SGD database. (C) The fraction of the 7,571 promoter regions (defined as in [B]) containing (green) or not containing (purple) a Z loci is shown. (D) H2A.Z is preferentially localized downstream of the NFR in yeast promoters. A sliding median of the ratio for H2A.Z/H2B (green) and H2A/H2B (gold) was plotted against the distance from the NFR (defined as a linker of more than 100 bp located less than 500 bp upstream of a 5′ gene boundary) for regions where the NFR can be unambiguously assigned to a single gene (i.e., NFR that map to two diverging genes were filtered out). A similar sliding window was applied to the nucleosome positioning data from Yuan et al. [37] (blue). A cartoon representation of the nucleosomal organization of a typical gene is shown at the bottom.

We next examined the actual location of the Z loci relative to genes. Manual inspection of the data suggests that Z loci are predominantly located within promoter regions. This feature is illustrated in Figure 1, that shows (i) only one ORF within the 40-KB window shown in Figure 1C contains enrichment for H2A.Z; (ii) none of the seven intergenic regions that do not contain any promoter in that region (for example, between *SOL2* and *ERS1*) contain a Z locus; (iii) there is a Z locus upstream of every ORF shown in Figure 1C; and (iv) interestingly, the intergenic region between the divergently transcribed *YCR090C* and *KIN82* ORFs contains two separate Z loci, supporting the idea that each promoter is decorated with its own H2A.Z nucleosome(s). In order to systematically evaluate the H2A.Z preference for promoter regions, we analyzed the relation between the H2A.Z/H2B ratio and the distance to the closest 5′ gene boundary (for example, the ATG for protein-coding genes) for all probes on the microarray. As shown in Figure 2A, H2A.Z binding (green line) is higher around the 5′ boundary with a small bias toward the regions located just upstream compared to downstream (see inlet for a zoom into the 5′ region). This relationship is not observed on random data (Figure 2A, black line) or when the proximity to the 3′ end of genes is examined (not shown). Because 5′ UTRs are very small in yeast, these data are consistent with H2A.Z being localized over promoters, although we cannot rule out the possibility that H2A.Z is rather present within the 5′ UTR since only a few transcriptional start sites have been annotated in yeast. For the sake of simplicity the word “promoter” will be used here to refer to the regions localized between 500 bp upstream and 100 bp downstream of the 5′ boundary of transcribed elements (this includes ORFs, but also other transcribed elements such as Ty elements, tRNAs, etc.). This interval was chosen based on a recent genome-wide analysis of yeast promoters [38]. As shown in Figure 2B, 74% (3,639 out of 4,862) of the Z loci identified in this study are located within the promoter of annotated genes. Considering that promoters account for about 31% of the yeast genome, this translates into a 2.4-fold enrichment over a random uniform distribution (*p* < 0.001). In addition, we find that 63% (4,742 out of 7,488) of yeast promoters are decorated with H2A.Z (Figure 2C). Conversely, only 2% of the H2A.Z loci are located within intergenic regions that contain no promoters (where two genes are transcribed convergently).

In a recent elegant study, the Rando group mapped nucleosomes over 482 KB of yeast DNA, including most of the entire Chromosome III. They showed that the scored yeast promoters contain a nucleosome-free region (NFR) flanked with well-positioned nucleosomes [37]. A median sliding window of their data is reproduced in Figure 2D (blue) along with our genome-wide location data with H2A.Z/H2B (green) and H2A/H2B (gold) data for a composite of 1,850 genes [37]. Despite both assays having different resolution, the figure clearly shows that H2A.Z is preferentially localized downstream of the NFR in yeast promoters. More important, data from the figure suggest that H2A.Z may occupy the first, and perhaps also the second, well-positioned nucleosome downstream of the NFR (see cartoon in Figure 2D). Taken together, these data suggest that H2A.Z targets specific nucleosomes within promoters across the genome and therefore contributes to create a promoter-specific chromatin structure.

In addition to class II promoter regions, we found H2A.Z loci at other transcribed units such as tRNAs, small nucleolar RNAs, rDNAs, and Ty elements, suggesting that the role of H2A.Z in transcription is not limited to protein-coding genes (data not shown). Interestingly, we also found H2A.Z loci centered around centromeres and replication origins (data not shown). The association of H2A.Z to centromeres was also reported previously [32], and the role of H2A.Z in DNA replication and chromosomal segregation will be studied elsewhere (Bataille et al., unpublished data).

### H2A.Z Is Not Enriched in the Promoter Region of Highly Active Genes

**Figure 3:**
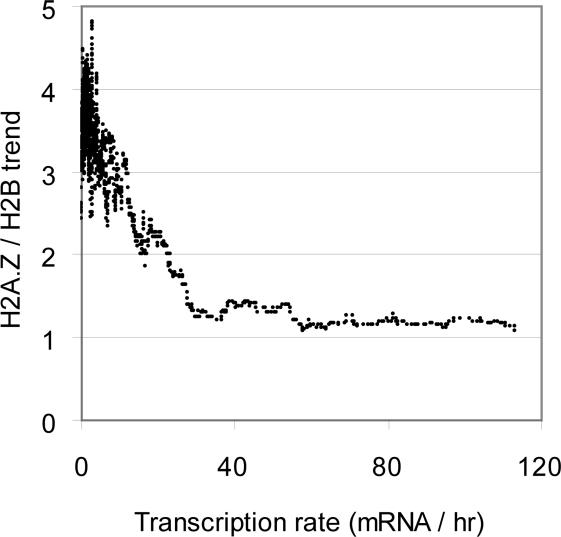
H2A.Z/H2B Ratios Are Inversely Correlated with Transcription Rate The binding trend was calculated by computing the moving median of the H2A.Z/H2B flat ratios over a sliding window of 50 genes across all genes ordered by transcription rate as described previously [36,40,41,60–62]. Each probe was assigned to its closest ATG, and the probe with the highest H2A.Z/H2B value for each gene was used. The transcription rate for yeast genes was determined previously [63].

Data from Figures 1 and 2 have clearly shown that H2A.Z has a preference for promoter regions. However, Figure 2B shows that up to 37% of promoters are not associated with a Z locus. In order to investigate what determines the association of H2A.Z with some promoters but not others, we investigated the correlation between H2A.Z promoter occupancy and the transcription rate for all genes. As shown in Figure 3, there is an inverse correlation between the transcription rate and H2A.Z occupancy. Accordingly, when the genome-wide location of H2A.Z was determined for cells grown in galactose, we noticed that the occupancy of H2A.Z was reduced, compared to its occupancy in glucose, at the promoters of 147 out of 173 genes whose expression levels have been demonstrated to be induced the most under those conditions according to the study of Ren et al. [39]. These data are consistent with H2A.Z being dynamically associated with inactive genes. These genomic observations are also in line with our ChIP/Q-PCR data (see Protocol S2 and Figure S2) and with previously published evidence at specific promoters [19,21,22]. We would like to point out that data represented in the experiment of Figure 3 were collected from a population of cells that are heterogeneous in the sense that each cell is in a different physiological state and phase of the cell cycle. For that reason, and also because promoters have inherently different strengths, a given gene is never completely off or on within the population. The observed transcription rate across all genes is therefore a continuum ranging from near zero to about 300 mRNA per hour for genes that are the most transcriptionally active. The transcription rate being a continuum, it is expected that the H2A.Z occupancy will also be a continuum. In that regard, RNA pol II or general transcription factors like TFIIB also show continuum-like binding patterns in those assays [36,40,41]. We also propose that the low enrichment of H2A.Z from the promoter of highly active genes most likely reflects the low abundance of nucleosomes in those regions since recent evidence shows that the promoters of highly active genes are depleted of nucleosomes [42–46]. Collectively, these data show that H2A.Z specifically associates with one or a few nucleosomes within the promoter of some inactive genes but is generally absent from the promoter of highly active genes.

### H2A.Z Regulates Nucleosome Positioning

**Figure 4:**
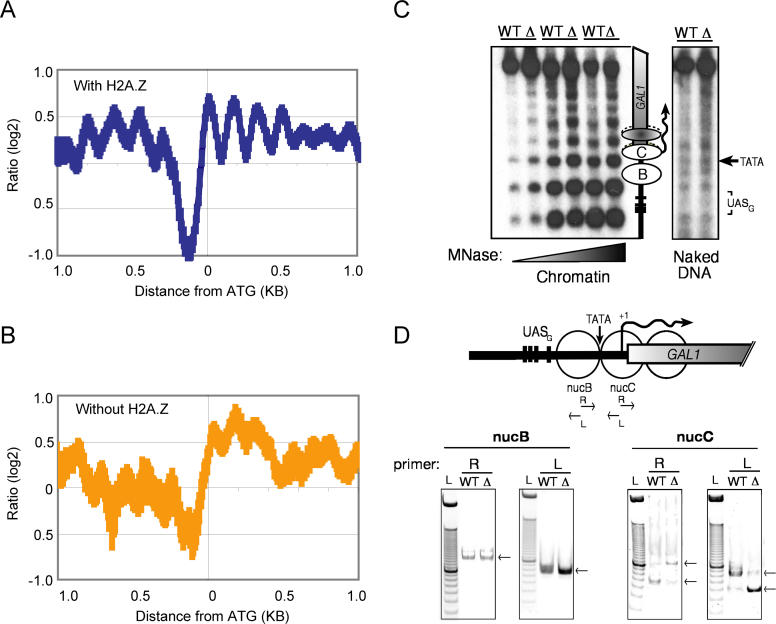
H2A.Z Regulates Nucleosome Positioning (A) Nucleosome positioning map of genes associated with a Z locus. The data from Yuan et al. [37] were used to compute the nucleosome occupancy curve for all genes containing a Z locus aligned on their ATG. Peaks represent nucleosomes, and valleys represent linker regions. An NFR is detected approximately 200 bp upstream of ATGs as described by Yuan et al. [37]. The vertical thickness of the curves contains 1-SD error bars for the mean log_2_ ratio. (B) Same as (A) but for genes containing no Z locus. (C) Indirect end-labeling of MNase-digested chromatin from WT and *htz1*Δ cells grown in the presence of glucose. (D) High-resolution LM-PCR analysis of MNase digested nucB–C mononucleosomes. Upper part: structure of the *GAL1* promoter and PCR probes used; left: nucB analysis probing the right (R), and left (L) boundaries in WT and *htz1*Δ (Δ) cells; right: same as left part of the figure, but the analysis is with nucC.

Having established that H2A.Z is present at most yeast promoters, we sought to investigate the functional significance of the promoter-specific localization of H2A.Z. First, we investigated nucleosome positioning of genes that have Z loci (Figure 4A) versus genes that are devoid of those loci (Figure 4B) as previously defined in Figure 2, using data presented in Yuan et al. [37]. The figure clearly shows that nucleosomes within the genes that bear Z loci are well positioned, whereas nucleosomes positioning at genes that do not have Z loci are significantly less well organized. This observation is consistent with the fact that H2A.Z-free promoters are less covered by nucleosomes than the more frequent H2A.Z-containing promoters (not shown). The figure further suggests that H2A.Z may play an important role in organizing chromatin structure at promoters. Alternatively, however, H2A.Z may associate with promoters as a consequence of their well-organized chromatin structure. In order to test directly whether H2A.Z can regulate chromatin structure at promoters, we studied nucleosome positioning at a model gene in wild-type (WT) and H2A.Z-null (*htz1*Δ) cells. The *GAL1* gene was chosen for these experiments, since H2A.Z is required for its full expression and since the variant histone preferentially binds the repressed promoter of that gene [19,21]. Furthermore, chromatin structure at the *GAL1* promoter has been thoroughly investigated [47–50], so any changes in chromatin structure should be experimentally tractable. First, we performed indirect end-labeling experiments on chromatin samples that were partially digested by MNase. Figure 4C, left panel, shows that the gross nucleosome positioning pattern is not significantly altered in *htz1*Δ cells when the latter are grown in media containing glucose. However, we do note a slight shift of nucleosome C (nucC) in a downstream position as well as a few other nucleosomes. The right panel of that figure shows the cleavage pattern of MNase-digested naked *GAL1* promoter DNA. The UAS_G_ region can be easily identified as it is quite resistant to MNase cleavage [49]. Since indirect end-labeling experiments only have a resolution of approximately 20–30 bp, we decided to carry out high-resolution LM-PCR assays of MNase-digested mononucleosomes at two positioned nucleosomes within the *GAL1* promoter region (nucB and nucC; see Figure 4D). Thus, LM-PCR linkers were ligated to mononucleosome-purified DNA, and PCR reactions were carried out with external oligonucleotides corresponding to the linkers in combination with oligonucleotides corresponding approximately to the center of the nucleosome-protected DNA as previously determined by Fisher-Adams and Grunstein [49]. These last oligonucleotides are designed to either amplify the right-end portion of nucleosome-embedded DNA (R) of nucB and nucC, or the left-end portion (L) (Figure 4D, above). The results show that nucB does not shift position in the *htz1*Δ mutant as compared to WT cells when PCR reactions were performed at either the right or left boundaries of the nucB. However, in the case of nucC, there is an approximately 20-bp shift to a downstream position in *htz1*Δ cells as compared to the WT case (Figure 4D, right panel). More important, this shift was observed with both the right and left boundaries of nucC. These results confirm our observations in indirect end-labeling experiments and suggest that the absence of H2A.Z induces a specific shift of nucC to a downstream position but has no effect on the positioning of nucB. Our results suggest that H2A.Z may be enriched at the promoters of inactive genes in order to modulate the position of key regulatory promoter-associated nucleosomes. Because we found that most Z loci are located within well-positioned nucleosomes and because H2A.Z-free promoters have a less organized chromatin structure than H2A.Z-containing genes, it is tempting to speculate that the variant histone also plays a role in nucleosome positioning at other genes.

### H2A.Z Is Unusually Localized at HZAD Genes

**Figure 5:**
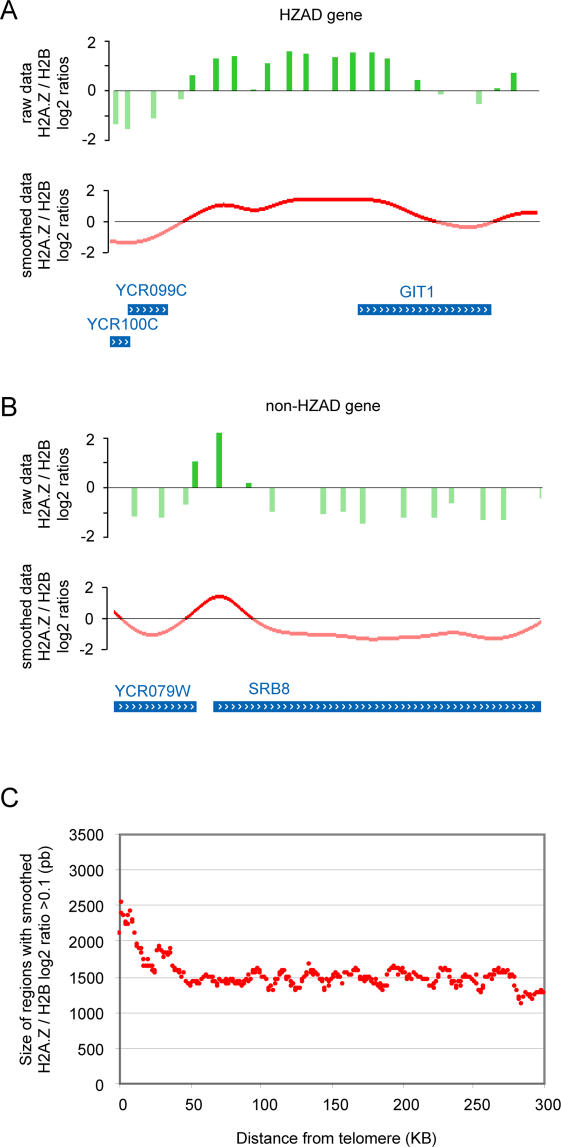
H2A.Z Is Unusually Localized in HZAD Genes (A) The raw (green) and smoothed (red) H2A.Z/H2B log_2_ ratios, as determined by our microarray experiment, are shown across a 5-KB region around the *GIT1* genes. The genes present in that region are shown in blue. (B) Same as (A) but for a 5-KB region around the *SRB8* gene. (C) H2A.Z covers wider regions near telomeres. The size of the regions covered by H2A.Z (as determined by distance between the coordinates where the smoothed H2A.Z/H2B log_2_ ratio reaches “0.1”) is plotted against the distance to telomeres. A moving average (window = 10 KB) was applied to the data.

Because genes contained within the so-called HZAD domains—unlike other H2A.Z regulated genes—are proposed to be regulated by H2A.Z through an antisilencing mechanism [24], we investigated more closely the distribution of H2A.Z at those genes. First, manual inspection suggested that H2A.Z occupied larger regions at those genes. As shown in Figure 5A, the regions occupied by H2A.Z around the *GIT1* gene (an HZAD gene) extend both upstream as well as downstream within the coding region as compared to a non-HZAD gene such as *SRB8* (see Figure 5B). Similar examples can be found in Figure S3. In order to investigate this in an unbiased manner, we have calculated the size of the regions localized between the coordinates where the H2A.Z occupancy curve approaches baseline (log_2_ ratio = 0.1). This size provides an estimation of the width of the Z loci. Figure 5C shows a plot of that size relative to the distance to chromosome ends. The figure shows that H2A.Z covers larger uninterrupted regions within the first 20 KB from telomeres compared to telomere distal regions. This is consistent with H2A.Z occupying wider regions around HZAD genes than at other genes in the genome. This conclusion was also reached using a different analysis described in Figure S4 and Protocol S3 and is not dependent on a difference in gene density at telomeres (not shown). This phenomenon is not restricted to HZAD genes since many telomere-proximal non-HZAD genes also show the same pattern (not shown). It is therefore possible that the antisilencing function observed at HZAD genes could actually be extended to more (perhaps most) telomere-proximal genes. Three possibilities could account for this wider distribution. First, H2A.Z could occupy more nucleosomes at those genes. Second, the number of nucleosomes occupied by H2A.Z may be the same, but these nucleosomes may not always be the same in different cells. Finally, it is also possible that chromatin at those genes is more fluid such that the H2A.Z nucleosomes can be found anywhere along a long stretch of DNA.

## Discussion

We have investigated the genome-wide distribution of histone variant H2A.Z as compared to the regular S-phase histone H2A. Our results show that H2A.Z can preferentially associate to narrow regions of yeast promoters. Surprisingly, the variant histone is present within most nucleosome-containing promoters. Conversely, the promoters of highly transcribed genes are not predominantly enriched in H2A.Z. Furthermore, and much to our surprise, H2A.Z does not appear to be as sharply localized within HZAD genes. The wider distribution of H2A.Z at HZAD genes may be important for preventing Sir protein spreading at those regions. Since H2A.Z shows a strong preference for inactive gene promoter regions, we also investigated whether the variant histone had an important role in modulating chromatin structure at the *GAL1* model gene. More important, we find that the replacement of H2A.Z for regular H2A in cells induces a shift in the positioning of a nucleosome located over the transcription start site. In accordance with this finding is the fact that nucleosomes at promoter regions bearing Z loci are well positioned as compared to genes not enriched in H2A.Z. Taken together, our results support a model where H2A.Z associates to specific nucleosomes in the promoters of inactive genes in order to poise, and perhaps organize, chromatin structure in a fashion that is permissive to transcription initiation. However, it remains to be determined whether the simple incorporation of H2A.Z into specific nucleosomes is by itself necessary and sufficient to facilitate gene activity or whether additional factors are required for that action. The first possibility is supported by the fact that in vitro-assembled chromatin arrays containing H2A.Z are inherently more resistant to condensation as compared to H2A-containing nucleosomal arrays [51,52]. On the other hand, work from our laboratory has shown that H2A.Z can interact with regulatory proteins and components of the transcriptional machinery [19], arguing that H2A.Z may regulate gene expression through interactions with such downstream effectors.

Although H2A.Z has been previously shown to be enriched at the *GAL1, PHO5,* and *PUR5* genes [21,22], the fact that H2A.Z globally associates to defined promoter regions of inactive genes is surprising since previous studies had suggested that even though H2A.Z was generally enriched in euchromatic regions, there did not appear to be any correlation between deposition of the variant histone and promoters [24,30]. However, we note that in those cases where H2A.Z was found more enriched in the ORF as compared to the promoter, the genes were highly transcribed housekeeping genes (e.g.,*ACT1* and *ADH1*) [24,30]. Recent studies have clearly established that the promoters of such highly active genes are rather depleted of nucleosomes [42–46], and thus the apparent low enrichment of H2A.Z at those promoters could actually reflect low nucleosome occupancy. In support of this conclusion, we find that H2B levels are significantly reduced at the *ACT1* promoter, while the H2A.Z/H2B ratio shows a slight enrichment in the promoter region (see Figure S2C). Because housekeeping genes are by definition transcribed in most, if not all, conditions, and since we found that H2A.Z is present at promoters of genes when they are inactive, we favor a model where H2A.Z would be more important for the regulation of nonhousekeeping genes that are generally repressed when grown in rich medium but are strongly induced under specific growth conditions (e.g.,*GAL* genes upon galactose induction, etc.). It is interesting to note that this last possibility is in line with published evidence regarding the role of H2A.Z in higher organisms. For example, H2A.Z is not significantly expressed in the nondifferentiated inner cell mass tissue of the mouse embryo, whereas it is highly expressed in the differentiating tissues [28]. Furthermore, H2A.Z is required for the proper development of *Xenopus* [13]. These observations may be taken to suggest that H2A.Z is only required when a particular gene expression program is engaged in an organism and not so much for gene expression that pertains to basic cellular metabolisms. Since H2A.Z has been shown to be abundantly associated with heterochromatin in higher eukaryotes [28]—which is much more preponderant in metazoans as compared to yeast [53,54]—it remains to be determined whether or not the variant histone plays additional functions linked to this localization in metazoans. It is worth emphasizing, however, that this colocalization of H2A.Z with metazoan heterochromatin does not exclude the possibility that it may also be associated with the promoter regions of inactive genes as we have shown with yeast. This possibility is currently under investigation.

Our genome-wide location assay has revealed that—as opposed to what is observed at other genes—H2A.Z occupies wider regions that spread out well beyond the promoter region and into the ORF, at HZAD genes. As previously suggested by Meneghini et al. [24], it is conceivable that H2A.Z has a dual role in gene expression, the first being that of poising genes for transcription initiation in euchromatin, and the second being that of protecting euchromatin from silencing. We propose that at most inactive genes where H2A.Z has a sharp promoter-binding pattern, H2A.Z will simply be incorporated into specific repressive nucleosomes that need to be remodeled quickly prior to gene induction, whereas at HZAD genes, H2A.Z would create nucleated domains that are refractory to gene silencing by heterochromatin. Alternatively to this scenario, it is possible that the broad distribution of H2A.Z at HZAD genes reflects unstable positioning of variant histone-bearing nucleosomes, perhaps due to a chromatin structure that is less well organized at those genes. This last possibility, however, is less likely since examination of the nucleosome positioning data from Yuan et al. [37] reveals that nucleosomes are well positioned over the Chromosome III-contained HZAD genes. Nevertheless, it is likely that the specific chromatin structure exemplified by this unusual localization of H2A.Z renders the HZAD genes more sensitive to Sir2 silencing.

Our observations that H2A.Z is also important in regulating nucleosome positioning, in particular at the *GAL1* promoter, may account for a mechanism by which H2A.Z regulates transcription. We had previously observed that absence of H2A.Z prevents RNA polymerase II and TBP from being efficiently recruited to the *GAL1* promoter, whereas the Gal4 activator was efficiently recruited to the gene [19]. In addition to these findings, recent experiments from our laboratory have shown that Mediator, SAGA, and Swi/Snf also cannot be efficiently recruited to *GAL1* in the absence of the variant histone (K. Lemieux and L. Gaudreau, unpublished data). We thus propose that improper positioning of at least one nucleosome over the *GAL1* transcription start site may be sufficient to severely impair the recruitment of the entire transcriptional machinery to the gene. Our results also emphasize the fact that chromatin architecture is exquisitely regulated, and even subtle changes in its structure may trigger transcription defects. A similar situation was recently reported with bromodomain factor 1 (Bdf1) and histone H4-tail acetylation at the *PHO5* gene. It was reported that a shift of 2–3 bp of a positioned nucleosome at a *PHO5-lacZ* reporter construct resulted in a defect in *PHO5* induction in the absence of Bdf1 and H4-acetylation [55]. This is of particular significance since Bdf1 is a component of Swr1.com that loads H2A.Z into chromatin [29,30]. It is conceivable that deletion of *BDF1* prevents loading of H2A.Z into chromatin, and therefore the Bdf1-related defect would be owed to improper loading of H2A.Z at the *PHO5* promoter. Alternatively, it is conceivable that Bdf1 could be a downstream target of H2A.Z and important in mediating its function in nucleosome positioning. These possibilities are currently under investigation. Finally, we point out that our data do not exclude the possibility that H2A.Z could also be important in transcription elongation since the latter histone variant has been shown to interact genetically with the TFIIS elongation factor in yeast [30] and since it appears to be replaced by H2A downstream of the *c-myc* promoter region during elongation [23].

## Materials and Methods

### Yeast strains and genetic methods

*Saccharomyces cerevisiae* W303a strains carrying 3xMyc-H2A.Z, 3xMyc-H2A or 3xHA-H2B were generated using PCR epitope tagging as described previously [56]. All *URA3* markers were “popped-out” and strains were verified by PCR and immunoblotting analyses. MAY424 (*htz1*Δ) was described previously [19].

### Antibodies

Antibodies in this study were from the following source: anti-Myc, (9E10; Santa Cruz Biotechnology, Santa Cruz, California, United States); anti-HA (12CA5; Roche, Basel, Switzerland); and anti-TFIIB, a kind gift from Richard A. Young.

### ChIPs

ChIP experiments were performed essentially as described previously [19,22] with some modifications. Briefly, 50 ml of cells were grown in yeast extract-peptone supplemented with 2% dextrose, or 2% raffinose/5% galactose for *GAL*-induced conditions, to an OD_600_ of 0.7 and fixed with 1% formaldehyde as described [19]. Next, 500 μl of whole-cell extract was incubated with the appropriate antibody coupled to magnetic beads (Dynal Biotech, Brown Deer, Wisconsin, United States). Immunoprecipitated DNA was either used for genome-wide location analysis (see below) or Q-PCR analysis. Briefly, immunoprecipitated DNA and input DNA were mixed with the appropriate oligonucleotides and Brilliant SYBR Green Q-PCR Master Mix (Stratagene) in 20 μl final volume. Q-PCR was performed on an Mx3000P Real-Time PCR System (Stratagene, La Jolla, California, United States). The sequences of the oligonucleotides used for all of the Q-PCR experiments are available in Table S7.

### DNA microarrays

The microarrays used for location analysis were purchased from Agilent Technologies (Palo Alto, California, United States). The microarrays contain a total of 44,290 60-mer probes (including 2,306 controls), covering the entire genome (except for repetitive regions) for an average density of one probe every 270 bp (±100 bp) within the probed regions. The greatest distance between the end of one probe and the beginning of the next is 540 bp, and 97% of the probes are within 350 bp of another one. The probes were mapped to the genome (UCSC) using BLAST. More than 99.9% of the probes mapped to a single locus, whereas 370 probes (<0.1%) had multiple BLAST outputs and were mapped accordingly. These microarrays were previously used to map histone modifications [57].

### DNA labeling and hybridization

Labeling was done as described before [58]. Briefly, the immunoprecipitated DNA fragments were blunted with T4 DNA polymerase and ligated to unidirectional linkers. The DNA was then amplified by LM-PCR in the presence of aminoallyl-modified dUTP. The labeling was carried out post-PCR using monoreactive Cy-dye NHS esters that react specifically with the aminoallyl-modified dUTP. Hybridization was performed as described in Pokholok et al. [57], using salmon sperm in place of herring sperm DNA. Detailed hybridization and labeling protocols can be found at http://www.ircm.qc.ca/microsites/francoisrobert/en.

### Data analysis

The data were normalized and replicates were combinined using a weighted average method as described previously [39]. To interpolate between probes and identify Z loci, a standard Gaussian filter (SD = 200 bp) was applied twice to the data. Z loci were defined as the maxima of this smoothed occupancy curve (excluding maxima smaller than zero).

### Chromatin analysis

*S. cerevisiae* nuclei were prepared as previously described [59]. Nuclei were washed once in digestion buffer (15 mM Tris-HCl [pH 8.0], 50 mM NaCl, and 1.4 mM CaCl_2_) and resuspended in 1.2 ml digestion buffer per gram of dry weight.

For indirect end-labeling experiments, resuspended nuclei were digested with either 0, 1, 3, 5, 25, or 50 U/ml of MNase (USB, Cleveland, United States). DNA was prepared as described [59]. Southern blot analysis was conducted using an EcoRI-BanII fragment from the *GAL1–10* region as a probe.

For LM-PCR nucleosome boundary mapping, 200-μl aliquots of resuspended nuclei were digested with a final concentration of 100 U/ml of MNase (USB) for 10 min at 37 °C and stopped with 4 mM EDTA. DNA was then prepared as described [59]. MNase-digested DNA was ligated overnight to unidirectional linkers, and mononucleosome-length fragments were then purified by agarose gel electrophoresis. PCR reactions were conducted with linker-specific and nucleosome-specific oligonucleotides, using JumpStart Taq DNA Polymerase (Sigma, St. Louis, Missouri, United States), using the manufacturer's recommendations. Oligonucleotide sequences are available in Table S8.

## Supporting Information

Figure S1 High-Resolution Mapping of H2A.Z at the SRB8 Locus by Q-PCR (A) Theory for domain size calculation using a control protein. A protein for which previous literature provides the actual size of the domain occupied by the protein on DNA (e.g., TFIIB) can be used to estimate the actual domain of an unknown protein (e.g., H2A.Z). Data from the control protein are used to estimate the amount of DNA that is added due to the limit of the resolution of the ChIP assay. This “resolution addition” is removed from the “observed” domain of the tested protein to generate its “actual” domain.
(B) Probes used in our Q-PCR assay. The regions amplified by PCR to probe our ChIP assay are depicted in red.
(962 KB TIF).

Figure S2 Q-PCR Analysis of H2A.Z ChIP Experiments (A) The locus including the *PRS2, UBC6, AST2,* and *RPS8B* genes is drawn to scale, and the positions of the PCR probes used to monitor H2A.Z occupancy are shown as black bars. The H2A.Z/H2B ratios are shown by gray bars.
(B) Same as in (A) but for the *GIT1* locus, a gene contained within an HZAD domain.
(C) Same as in (A) and (B) but for the *ACT1* locus. The H2B/input ratio is also shown as white bars. In each panel, the probe with the lowest ratio was set to “1.”
(763 KB TIF).

Figure S3 H2A.Z Occupancy at HZAD10 (A) The raw (green) and smoothed (red) H2A.Z/H2B ratios are shown across a 15-KB region from Chromosome IX containing HZAD10 (gray shaded box). The genes in that region are shown in blue.
(B) Same as in (A) but for a non-HZAD region from Chromosome III.
(749 KB TIF).

Figure S4 Z Loci Are Wider around Telomeres (A) Graphical representation of the width index calculation.
In order to look at the spread of the H2A.Z domains across chromosomes we have calculated an index estimating the relative width of these domains. The width index (i) is defined as *x/y,* where *x* is the width observed at ½ *y, y* being the height at the maximum of the H2A.Z smooth occupancy curve. The width is divided by the height in order to normalize for the fact that higher peaks will necessarily take more space to reach baseline and will therefore look like wider domains than would small peaks.
In order to avoid “contamination” by overlapping peaks, maxima localized within less than 1.5 KB from one another were not considered in the analysis.
(B) The width trend was determined by computing a sliding median of the width index (see A) across all yeast genes ordered by their distance from the chromosomal end and plotted against that distance.
(265 KB TIF).

Protocol S1 Determination of the Size of the Z Locus within the *SRB8* Promoter (27 KB DOC).

Protocol S2 Validation of ChIP–Chip Data by Q-PCR (26 KB DOC).

Protocol S3 Z loci Are Wider around Telomeres (24 KB DOC).

Table S1 Raw Data for the H2A.Z/H2B, H2A/H2B, and H2A.Z/H2A.Z ChIP–Chip Experiments For each experiment, the normalized enrichment ratio is shown for every probe on the microarray.
(4.3 MB ZIP).

Table S2 Smoothed Data for the H2A.Z/H2B ChIP–Chip Experiment For every coordinate within the genome, the H2A.Z/H2B ratio derived from the raw data by the Gaussian plot analysis is shown. This file is a text file ready for display within the UCSC Genome Browser.
(10.8 MB ZIP).

Table S3 Smoothed Data for the H2A/H2B ChIP–Chip Experiment For every coordinate within the genome, the H2A/H2B ratio derived from the raw data by the Gaussian plot analysis is shown. This file is a text file ready for display within the UCSC Genome Browser.
(10.5 MB ZIP).

Table S4 Smoothed Data for the H2A.Z/H2A.Z ChIP–Chip Experiment For every coordinate within the genome, the H2A.Z/H2A.Z ratio derived from the raw data by the Gaussian plot analysis is shown. This file is a text file ready for display within the UCSC Genome Browser.
(10.6 MB ZIP).

Table S5 List of Z Loci A table listing the position of each Z loci identified in this study.
(340 KB XLS).

Table S6 Genome Browser-Ready File A BED file ready for displaying the H2A.Z/H2B raw (green) and smoothed (red) data for the entire yeast genome as in Figure 1.
(11.2 MB ZIP).

Table S7 Sequences of the Oligonucleotides Used to Quantify ChIPs by PCR in This Study (28 KB XLS).

Table S8 Sequences of the Oligonucleotides Used in the LM-PCR Assay to Map Nucleosomes within the *GAL1* Promoter (18 KB XLS).

### Accession Numbers

The *Saccharomyces* Genome Database (http://www.yeastgenome.org/) accession numbers for the genes and proteins discussed in this paper are *ACT1* (SGDID S000001855), *ADH1* (SGDID S000005446), Bdf1 (SGDID S000004391), *ERS1* (SGDID S000000671), *GAL1* (SGDID S000000224), Gal4 (SGDID S000006169), *GIT1* (SGDID S000000695), *KIN82* (SGDID S000000687), *PHO5* (SGDID S000000297), *PUR5* (SGDID S000001259), Sir2 (SGDID S000002200), *SOL2* (SGDID S000000718), *SRB8* (SGDID S000000677), Swr1 (SGDID S000002742), TBP (SGDID S000000950), TFIIB (SGDID S000006290), and *YCR090C* (SGDID S000000686).

## Abbreviations

Bdf1: bromodomain factor 1
ChIP: chromatin immunoprecipitation
ChIP–chip: chromatin immunoprecipitation coupled with tiling microarray analysis
IP: immunoprecipitate
LM-PCR: ligation-mediated PCR
nucC: nucleosome C
NFR: nucleosome-free region
ORF: open reading frame
Q-PCR: quantitative PCR
