# Supplementary material for: Variant Histone H2A.Z Is Globally Localized to the Promoters of Inactive Yeast Genes and Regulates Nucleosome Positioning
Source: PLoS Biol. 2005 Nov 1;3(12):e384. doi: 10.1371/journal.pbio.0030384 (PMC1275524; doi:10.1371/journal.pbio.0030384)
Supplement: Supplementary file 6 [file pbio.0030384.sd001.doc]

**Supporting text 1**

**Determination of the size of the Z locus within the *SRB8* promoter**

The size of the observed domain bound by a given protein, as determined by ChIP assays, is necessarily larger than the actual domain, due to the resolution limit of the technique. The factors that determine the observed size of the domain include the size of the chromatin fragments used in the IP, the size of the PCR fragments used to probe the ChIP reactions, and the distance between these probes. When these parameters are well established, models can be used to calculate the actual size of a domain from the observed data [1]. Alternatively, the size of the domain can be estimated by comparing the observed domain with that of a protein for which the actual domain is known [2] (See Figure S1A). This last strategy was used here to map H2A.Z at the *SRB8* locus. The data is shown in Figure 1D and more details are provided below.

In order to map the domain occupied by H2A.Z around the *SRB8* gene we designed 25, ~150bp-long, overlapping PCR probes covering a ~2.5KB region around the *SRB8* promoter. These probes were used in Chromatin Immunoprecipitation (ChIP) experiments to map H2A.Z and TFIIB at that region. A schematic representation of the probes is shown in Figure S1B and oligonucleotide sequences can be found in TableS7. Since the observed domain occupied by H2A.Z is ~250 bp larger that the one observed for TFIIB (see Figure 1D), we can infer that H2A.Z occupied a domain of about 260 bp at this locus. This data is consistent with our ChIP-chip data and provides a higher-resolution map of H2A.Z at the *SRB8* gene.

1. Kadosh D, Struhl K (1998) Targeted recruitment of the Sin3-Rpd3 histone deacetylase complex generates a highly localized domain of repressed chromatin in vivo. Mol Cell Biol 18: 5121-5127.
2. Ng HH, Robert F, Young RA, Struhl K (2003) Targeted Recruitment of Set1 Histone Methylase by Elongating Pol II Provides a Localized Mark and Memory of Recent Transcriptional Activity. Molecular Cell 11: 709-719.
