# Supplementary material for: Variant Histone H2A.Z Is Globally Localized to the Promoters of Inactive Yeast Genes and Regulates Nucleosome Positioning
Source: PLoS Biol. 2005 Nov 1;3(12):e384. doi: 10.1371/journal.pbio.0030384 (PMC1275524; doi:10.1371/journal.pbio.0030384)
Supplement: Supplementary file 7 [file pbio.0030384.sd002.doc]

**Supporting text 2**

**Validation of ChIP-chip data by Q-PCR**

Many loci enriched in H2A.Z according to our ChIP-chip assay were validated by Q-PCR. Figure S2 shows Q-PCR data for these loci. Figure S2A shows that the H2A.Z/H2B ratio is significantly higher in the *PRS2* (SGD:S000000901)*-UBC6* (SGD: S000000902) and *AST2* (SGD: S000000903)*-RPS8B* (SGD: S000000904) intergenic regions than in the ORFs and at the putative terminator region abutting *UBC6* and *AST2*. The figure also shows that the H2A.Z/H2B ratio peaks rather sharply in promoter regions. Interestingly, the promoter of the highly transcribed *RPS8B* gene is devoid of H2A.Z. The case of highly transcribed genes is discussed below. Taken together, these data fully support our conclusion that H2A.Z preferentially targets the promoter region of inactive yeast genes.

Figure S2B shows Q-PCR analyses of ChIP experiments at the *GIT1* locus, a gene contained within a HZAD domain. In full support to our ChIP-chip data, the Q-PCR analysis depicted here shows that H2A.Z occupies a wider region at that gene compared with non-HZAD genes like those shown in panel A.

Figure S2C shows Q-PCR analysis of the *ACT1* gene. The *ACT1* gene is highly transcribed and previous study had implied that H2A.Z levels were higher at that ORF relative to the promoter of that gene. In this same study, however, the level of H2A.Z was not normalized to nucleosome density. Since recent studies have clearly established that the promoters of such highly active genes are depleted of nucleosomes [1-5], the apparent low enrichment of H2A.Z at those promoters could actually reflect low nucleosome occupancy. In support of this conclusion, Figure S2C shows that H2B levels are significantly reduced from the *ACT1* promoter, while H2A.Z/H2B binding shows enrichment in the promoter region relative to the ORF.

1. Lee CK, Shibata Y, Rao B, Strahl BD, Lieb JD (2004) Evidence for nucleosome depletion at active regulatory regions genome-wide. Nat Genet 36: 900-905.

2. Reinke H, Horz W (2003) Histones are first hyperacetylated and then lose contact with the activated PHO5 promoter. Mol Cell 11: 1599-1607.

3. Schwabish MA, Struhl K (2004) Evidence for eviction and rapid deposition of histones upon transcriptional elongation by RNA polymerase II. Mol Cell Biol 24: 10111-10117.

4. Boeger H, Griesenbeck J, Strattan JS, Kornberg RD (2003) Nucleosomes unfold completely at a transcriptionally active promoter. Mol Cell 11: 1587-1598.

5. Boeger H, Griesenbeck J, Strattan JS, Kornberg RD (2004) Removal of promoter nucleosomes by disassembly rather than sliding in vivo. Mol Cell 14: 667-673.
