# Supplementary material for: Variant Histone H2A.Z Is Globally Localized to the Promoters of Inactive Yeast Genes and Regulates Nucleosome Positioning
Source: PLoS Biol. 2005 Nov 1;3(12):e384. doi: 10.1371/journal.pbio.0030384 (PMC1275524; doi:10.1371/journal.pbio.0030384)
Supplement: Supplementary file 8 [file pbio.0030384.sd003.doc]

**Supporting text 3**

**Z loci have a wider distribution pattern around telomeres**

The data presented in Figure 5C suggests that Z loci have a wider distribution pattern within the first 20 KB from telomeres compared to telomere-distal regions. The possibility remain, however, that peaks close to telomeres appear wider in this analysis simply because they are taller (tall peaks will necessarily appear wider) or because telomere proximal regions contain a higher density of peak that would result in peak merging (that would look like a single wide peak in the analysis). In order to control for these possibilities, we computed a width index for all Z loci. Figure S4A provides a detailed explanation of this index. In sum, the width index is a measure of the width of the Z loci that is normalized for peak height. We also removed peaks that were close to each other (<1.5KB on each side) in order to avoid overlapping peaks that may contribute to the observed width of its neighbors. Figure S4B shows that this width index is larger (meaning wider Z loci) within the first 20KB from telomeres compared to telomere-distal regions. This is consistent with the data shown in Figure 5C and confirms that H2A.Z occupies wider regions around HZAD genes than at other genes in the genome and that is not due to a higher peak density or to taller peaks.
